# The Impact of Heavy Metals Exposure on Diabetic Foot Complications: A Systematic Review of Development, Progression, and Wound Healing

**DOI:** 10.3390/toxics14080690

**Published:** 2026-08-04

**Authors:** Richard O. Machava, Bheki T. Magunga, Simiso M. Ntuli, Thokozani P. Mbonane

**Affiliations:** 1 Department of Environmental Health, Faculty of Health Sciences, University of Johannesburg, Johannesburg 2006, South Africa; rmachava@uj.ac.za (R.O.M.); bhekim@uj.ac.za (B.T.M.); 2 Department of Podiatry, Faculty of Health Sciences, University of Johannesburg, Johannesburg 2006, South Africa; sntuli@uj.ac.za

**Keywords:** diabetic foot ulcer, heavy metals, environmental toxicology, wound healing, cadmium, lead, chelation therapy, oxidative stress

## Abstract

Diabetic foot ulcers (DFUs) constitute a significant global health burden; however, the influence of environmental heavy metals toxicity on their pathogenesis remains insufficiently investigated, particularly within low- and middle-income countries. This systematic review, conducted in strict accordance with PRISMA 2020 guidelines, assesses the impact of heavy metals exposure on the development, progression, and wound-healing kinetics of DFUs. A comprehensive search across PubMed/MEDLINE, Embase, Scopus, and Web of Science identified 32 eligible original studies. Quality appraisal was performed using the Newcastle-Ottawa Scale and SYRCLE’s Risk of Bias tool. Synthesized epidemiological and toxicological data consistently indicate that chronic exposure to non-essential heavy metals, specifically cadmium and lead, significantly augments DFU prevalence and clinical severity, demonstrating a 64% increase in DFU prevalence for each 1 µg/L increase in blood cadmium concentration. Mechanistically, these xenobiotics induce a ‘chronic inflammatory lock’ characterized by severe oxidative stress, sustained NLRP3 inflammasome activation, and the suppression of protective Metallothionein 2A (MT2A) pathways, thereby impeding microvascular angiogenesis. Conversely, deficiencies in essential trace elements, such as zinc and selenium, actively impair extracellular matrix maintenance. Advanced therapeutic interventions, including metal-ion-releasing hydrogels, cell-free exosome therapies, and systemic EDTA chelation, demonstrate significant potential to counteract heavy-metal-induced cellular suppression and facilitate tissue repair. This review underscores the imperative of integrating environmental toxicant screening and targeted detoxification strategies into standard multidisciplinary diabetic foot management.

## 1. Introduction

Diabetes mellitus constitutes a preeminent and escalating global public health challenge of the 21st century, imposing substantial clinical and economic burdens on health systems worldwide [1,2]. Among its most debilitating clinical manifestations is diabetic foot disease, principally underpinned by peripheral neuropathy and peripheral arterial disease (PAD). This complex pathological state frequently culminates in chronic, non-healing.

Diabetic foot ulcers, severe secondary infections, and, ultimately, lower-extremity amputations [3]. While traditional risk factors, such as suboptimal glycemic control, prolonged disease duration, mechanical stress, and dyslipidemia, are extensively documented, these clinical parameters do not fully elucidate the considerable heterogeneity observed in ulcer development rates and the pronounced impairment of wound healing across diverse patient populations [4,5,6]. Consequently, identifying novel, modifiable environmental determinants that accelerate these pathogenic pathways is crucial for advancing secondary prevention strategies. Concurrently, chronic environmental exposure to heavy metals and metalloids, specifically lead, cadmium, arsenic, and mercury, has emerged as a significant systemic driver of metabolic and vascular dysfunction [7]. Due to rapid industrialization, agricultural chemical runoff, and persistent environmental contamination, human exposure to these non-biodegradable toxic elements remains pervasive. Once absorbed, these heavy metals exhibit lengthy biological half-lives, bioaccumulating extensively within critical organ systems [8]. While the direct diabetogenic potential of specific toxic metals via pancreatic *β*-cell destruction is well-established, their compounding impacts on existing secondary microvascular and macrovascular complications remain inadequately elucidated in the current literature. Emerging toxicological evidence indicates that heavy metal accumulation profoundly exacerbates the clinical trajectory of diabetes, transforming otherwise manageable metabolic imbalances into severe, limb-threatening vascular pathologies [9,10,11]. The convergence of toxic metal exposure and diabetic foot disease pathophysiology is mediated by multiple, interconnected mechanisms. At the molecular level, heavy metals act as potent initiators of cellular damage, substantially augmenting the generation of reactive oxygen species and inducing profound systemic oxidative stress [8,12,13]. Within a diabetic milieu, this metal-induced oxidative stress accelerates the formation and accumulation of advanced glycation end-products, ultimately leading to the progressive cross-linking of dermal collagen, structural arterial stiffening, and profound endothelial dysfunction [14,15,16,17]. Furthermore, metals such as lead and cadmium attenuate the bioavailability of endothelial nitric oxide (NO), thereby impairing critical vasodilation and exacerbating the localized lower-limb ischemia characteristic of peripheral artery disease. Concurrently, the neurotoxicity of these metals induces damage to the myelin sheath and impedes axonal nerve conduction velocity, thereby accelerating the onset of insensate peripheral neuropathy [18,19,20,21]. This neuropathy serves as a primary precursor to unnoticed mechanical trauma and subsequent pedal ulceration.

Subsequent to ulcer formation, heavy metal toxicity profoundly disrupts the intricately coordinated, multi-phasic physiological cascade essential for successful dermal wound healing [22,23]. As comprehensively detailed in the recent literature on fundamental wound healing mechanisms, efficient wound closure necessitates a heavily orchestrated and overlapping sequence of hemostasis, inflammation, cellular proliferation, angiogenesis, and tissue remodelling [24,25]. Heavy metals significantly perturb this temporal sequence by disrupting immune cell functions, including macrophage polarization and neutrophil activity, thereby perpetuating a prolonged, hyper-inflammatory state within the wound bed that impedes tissue repair [26,27,28]. Additionally, toxic metals inhibit the migration and proliferation of dermal fibroblasts and keratinocytes, while simultaneously suppressing vascular endothelial growth factor signalling, consequently impeding critical capillary neo-angiogenesis [29]. This microvascular failure results in a local wound bed that remains profoundly ischemic, hypoxic, and highly vulnerable to aggressive, opportunistic bacterial infections refractory to conventional clinical interventions.

Despite the growing body of experimental research detailing these toxicological mechanisms, a critical gap remains in synthesizing this evidence into a cohesive clinical and epidemiological framework. Existing systematic reviews have primarily focused either broadly on the metabolic impacts of heavy metals on general glycemic control or exclusively on the environmental aetiology of peripheral vascular diseases, without integrating how these environmental toxins influence the localised, multi-systemic pathology of the diabetic foot [7,30,31,32,33]. To address this gap, this systematic review rigorously evaluates the comprehensive impact of environmental heavy metal exposure on the development, progression, and wound-healing trajectories of diabetic foot complications. By integrating toxicological pathways with established clinical parameters, this study aims to elucidate the hidden environmental risks affecting vulnerable diabetic cohorts and provide evidence-based insights to inform future public health policies and targeted environmental interventions.

## 2. Materials and Methods

This systematic review followed PRISMA 2020 guidelines. The protocol, based on Systems Thinking and One Health, linked environmental chemical exposure data to pathophysiological and clinical outcomes in metabolic diseases. It was prospectively registered in PROSPERO, CRD420261413223.

### 2.1. Search Strategy and Database Sources

The systematic literature search was conducted across four biomedical and environmental databases: PubMed/MEDLINE, Embase (Elsevier), Scopus (Elsevier), and Web of Science (Clarivate Analytics). To cover both legacy toxicological data and emerging molecular research, the search included literature from the database’s inception through May 2026. No geographic or language filters were applied; non-English articles were translated to avoid selection bias. Search syntax was developed using Medical Subject Headings (MeSH), Embase Emtree terms, free-text keywords, and Boolean operators (AND, OR). The search string was structured into three thematic blocks representing the review’s core domains: (1) heavy metals and toxic elements, (2) diabetes mellitus and metabolic dysfunction, and (3) foot complications or wound-healing outcomes. Specific Boolean search strings for each database are detailed below. The baseline configuration was:

(“heavy metal” OR “toxic metal” OR “toxic element” OR “lead” OR “Pb” OR “cadmium” OR “Cd” OR “arsenic” OR “As” OR “mercury” OR “Hg” OR “environmental pollutant”) AND (“diabetes mellitus” OR “diabetic” OR “hyperglycemia” OR “type 2 diabetes”) AND (“diabetic foot” OR “foot ulcer” OR “peripheral neuropathy” OR “diabetic neuropathy” OR “peripheral arterial disease” OR “PAD” OR “wound healing” OR “cicatrization” OR “ischemia”)

To maximise search sensitivity, electronic database searches were supplemented by manual reference screening (“snowballing”) of identified systematic reviews, meta-analyses, and textbook chapters. Grey literature was searched on OpenGREY and ProQuest Dissertations and Theses Global to mitigate publication bias.

### 2.2. Eligibility Criteria and Inclusion

To ensure a transparent, reproducible, and objective literature selection process, studies were screened and selected based on explicitly defined eligibility criteria, which were established using an adapted PECOS (Population, Exposure, Comparator, Outcome, and Study Design) framework:

- Population (P): Human cohorts of any age or location with confirmed Type 1 or Type 2 Diabetes Mellitus. Also included were in vivo mammalian and in vitro cell-line models demonstrating microvascular, macrovascular, or cellular mechanisms generalizable to diabetic dermal, vascular, or neurological tissues.
- Exposure (E): Primary exposure was chronic or acute environmental or occupational heavy metals or metalloids: cadmium, lead, arsenic, and nickel. Exposure quantification required validated analytical methods (e.g., Inductively Coupled Plasma Mass Spectrometry (ICP-MS), Atomic Absorption Spectroscopy (AAS) in human biological matrices (e.g., whole blood, urine, hair, nails, tissue biopsies) or environmental matrices (e.g., potable water, top-soil, ambient air modelling).
- Comparator (C): Eligible studies included a control group or comparison cohort with low, baseline, reference-level, or undetectable concentrations of targeted heavy metals. Experimental models used non-exposed control lines or vehicles.
- Outcomes (O): Studies reported at least one primary or secondary outcome. Primary outcomes included incidence, prevalence, relative risk, or severity of diabetic foot ulcers (DFUs), peripheral neuropathy, or peripheral arterial disease. Secondary outcomes included dermal wound healing kinetics, wound closure velocity, cellular proliferation, microvascular capillary neo-angiogenesis, or changes in metal-binding and immune-transporting gene expressions (e.g., *MT2A*, *GK*, *SLC31A1*, *ADNP*).
- Study Design (S): Eligible study designs included original, peer-reviewed, full-text research: prospective and retrospective cohort studies, case–control studies, cross-sectional epidemiological designs, and experimental in vivo or in vitro mechanistic toxicological studies.

### 2.3. Study Selection and Screening

The articles extracted were exported to EndNote 21 (Clarivate Analytics, Philadelphia, PA, USA) for centralized management and systematic deduplication. The deduplicated dataset was then uploaded to Covidence systematic review software (Veritas Health Innovation) for independent screening. Two blind reviewers (R.M. and B.M.) independently screened titles and abstracts in duplicate against pre-established inclusion criteria. Records meeting these initial eligibility thresholds advanced to full-text review, which was similarly conducted in duplicate by the three reviewers. Inter-reviewer discrepancies regarding study inclusion at either the abstract or full-text screening stages were resolved through structured consensus discussions. If a consensus could not be reached, two senior reviewers (S.M. and T.P.M.) acted as arbitrators. The entire selection progression, including explicit reasons for full-text exclusions, was documented in a formal PRISMA flowchart.

### 2.4. Data Extraction and Management

Data extraction was performed using a standardized, pilot-tested Microsoft Excel form. To ensure accuracy and minimize errors, two investigators (R.O.M. and B. T. M.) independently extracted data in duplicate. Extracted data comprised: (1) bibliographic characteristics (author, year of publication, country, and journal), (2) epidemiological and experimental design (sample size, cohort characteristics, follow-up duration, and cell-line or animal model specifications); (3) exposure assessment (type of metal, analytical detection method [e.g., ICP-MS or AAS], and exposure concentrations or biomarker levels), (4) clinical and physiological outcomes (incidence of neuropathy, peripheral artery disease, or ulceration; wound-healing rates; and associated molecular markers), and (5) statistical measures (Odds Ratios [OR], Relative Risks [RR], Hazard Ratios [HR], or Beta coefficients with corresponding 95% Confidence Intervals [CI] and *p*-values).

### 2.5. Quality and Risk of Bias Assessment

Quality and risk of bias were assessed, stratified by study design, utilizing the Newcastle-Ottawa Scale (NOS) for human epidemiological studies and SYRCLE’s Risk of Bias tool for in vivo animal models. Table 1 presents the Newcastle-Ottawa Scale quality appraisal for included human epidemiological studies. Overall, these observational studies exhibited moderate to high quality. Specifically, the cohort and cross-sectional designs demonstrated moderate-to-high methodological quality, scoring between 5 and 9 stars. The primary risk of bias stems from limited adjustment for environmental co-exposures and confounders.

Laboratory animal models demonstrated a low risk of bias in baseline characteristics; however, an overall high or unclear risk of bias was evident in allocation concealment and investigator blinding. Table 2 presents the risk of bias for in vivo toxicological studies, as assessed using the SYRCLE tool. It indicates a low risk of bias concerning incomplete outcome data and standard housing. However, performance and detection biases remain largely high or unclear, primarily attributed to prevalent shortcomings in investigator blinding and allocation concealment protocols.

## 3. Results

### 3.1. Study Selection and Selection Flow

A systematic database search of PubMed/MEDLINE, Embase, Scopus, and Web of Science identified an initial total of 418 citations. After removing 142 duplicate records (118 identified through automated deduplication and 24 through manual verification), the remaining 276 unique titles and abstracts underwent independent screening by two blinded reviewers (R.O.M. and B.T.M.). This primary screening phase resulted in the exclusion of 211 records that did not satisfy the predefined PECOS eligibility criteria.

Subsequently, 65 full-text articles were comprehensively assessed for eligibility. Of these, 33 full-text articles were excluded based on predefined methodological criteria, specifically:

- incorrect study design or absence of original data (n = 14),
- irrelevant exposure metrics not incorporating quantified heavy metal biomarkers (n = 9),
- omission of secondary diabetic foot or wound healing outcomes (n = 7) and
- overlapping cohort data reporting (n = 3).

**Figure 1:**
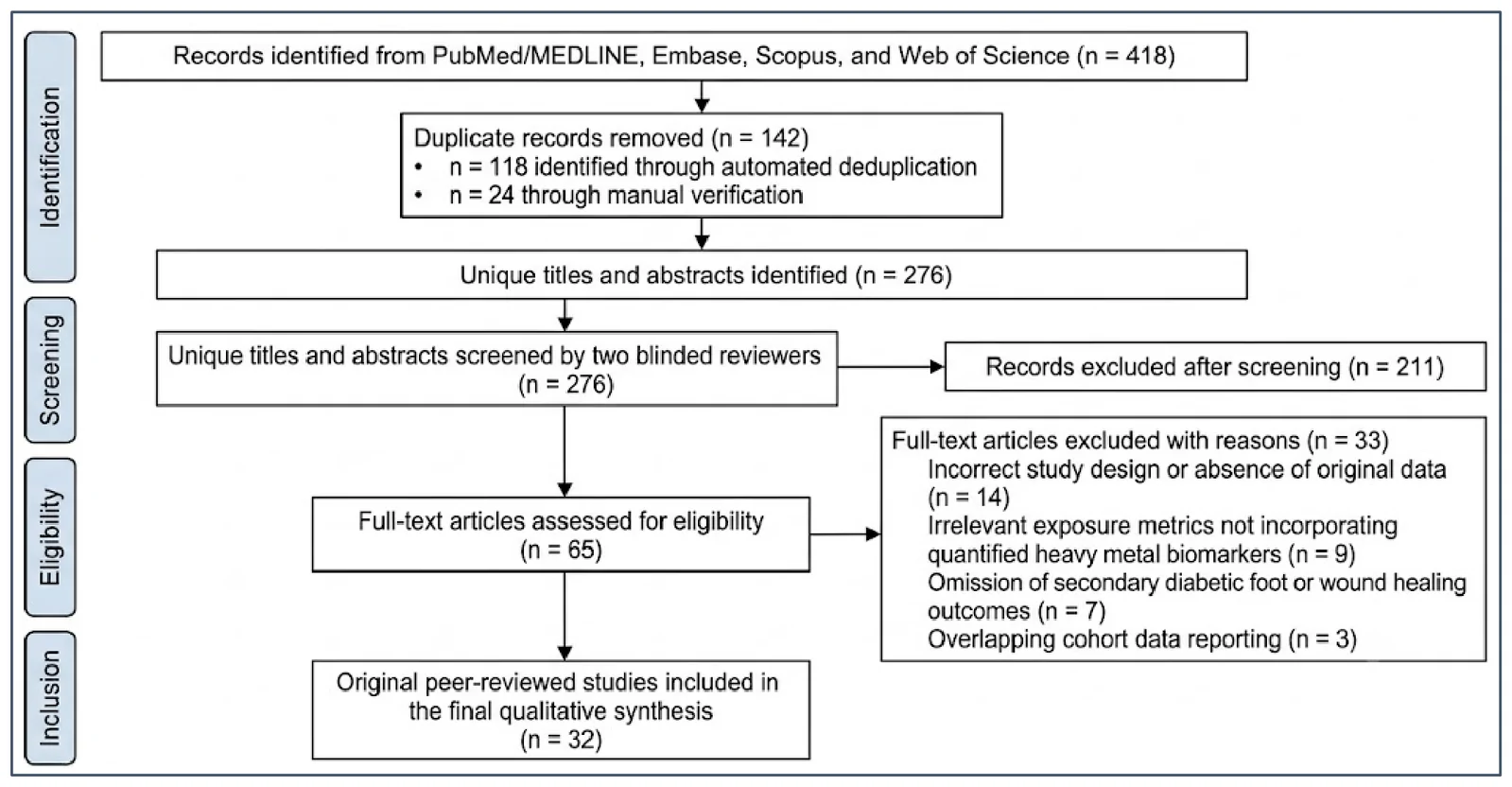
PRISMA flowchart.

Consequently, 32 original peer-reviewed studies met all eligibility criteria and were thus included in the final qualitative synthesis, as shown in Figure 1.

### 3.2. Clinical and Epidemiological Findings

The clinical and epidemiological studies included in this review reveal a clear distinction between the pathogenic risks associated with environmental toxic heavy metals and the essential roles of trace elements in the pathogenesis of diabetic foot ulcers (DFUs). Population-level cohort and cross-sectional data demonstrate that chronic exposure to cadmium in the environment is a significant risk factor for developing DFUs. These studies show a direct linear dose–response relationship, indicating that a 1 µg/L increase in blood Cd concentration corresponds to a 64% increase in the prevalence of DFUs.

Similarly, epidemiological findings consistently identify elevated blood lead and nickel concentrations in diabetic cohorts with active foot complications compared to non-ulcerated diabetic controls. In these human cohorts, chronic exposure to heavy metals is linked to systemic metabolic disruption and impaired glycemic control, which in turn affects tissue integrity. On the other hand, clinical observational data suggest that systemic deficiencies in essential trace elements, particularly zinc and selenium, are strongly associated with a higher incidence and faster onset of severe DFUs.

### 3.3. Experimental and Mechanistic Findings (In Vivo and In Vitro Models)

Although clinical and epidemiological trends indicate that environmental exposure to heavy metals is a systemic risk factor for DFU development, a closer examination of the underlying cellular disruptions through controlled experimental models is needed to understand this clinical progression. Toxicological data from in vivo mammalian models and in vitro cell-line studies have identified a complex network of cellular pathways through which heavy metals accelerate tissue damage, leading to the progression from minor tissue trauma to severe, deep-tissue complications.

In these experimental models, non-essential toxic metals such as cadmium, lead, and nickel act as potent inducers of cellular damage, resulting in the excessive production of reactive oxygen species and subsequent severe localized lipid peroxidation. This ongoing oxidative stress triggers the sustained upregulation of Nuclear Factor-kappa B (NF-κB) and activates the NLRP3 inflammasome pathways. This continuous molecular signalling leads to the release of pro-inflammatory cytokines, including interleukin-6 (IL-6) and tumour necrosis factor-alpha (TNF-α). Additionally, in in vivo wound models at the dermal level, severe tissue damage is characterized by a notable decrease in the expression of Metallothionein 2A (MT2A), a transcriptional downregulation that directly correlates with the development of highly refractory, non-resolving ulcers.

### 3.4. Impact on the Dermal Wound-Healing Cascade and Advanced Therapeutics

This systematic review underscores the significant impact of systemic heavy metals accumulation on the efficacy of multi-phasic dermal wound healing, underscoring the need for advanced therapeutic interventions. It is crucial to differentiate between interventions supported by clinical evidence and those proposed as theoretical or pre-clinical advancements.

The collective clinical data from the analyzed cohorts demonstrate that rectifying trace element deficiencies and eliminating toxic burdens are vital clinical interventions. Managing systemic zinc deficiencies, particularly in severe Wagner-grade ulcers, through oral zinc supplementation enhances metabolic parameters and expedites wound closure. Replenishing selenium levels, especially in conjunction with vitamins C and E, effectively reduces systemic oxidative stress markers. For individuals with elevated heavy metal levels, systemic chelation therapies such as intravenous Edetate disodium (EDTA) substantially decrease toxic metal concentrations, facilitating limb preservation and augmenting the population of healthy circulating progenitor cells, see Table 3 for more interventions.

To surmount impediments to healing and heavy metal-induced cellular inhibition, advanced bioengineering and regenerative interventions are proposed as potential strategies. Advanced bioengineering encompasses hydrogels that release copper ions and polymeric wound dressings containing silver and gold nanoparticles, offering localized antimicrobial effects without systemic toxicity. Elevated levels of lead and cadmium in tissues compromise stem cell metabolism, diminishing the regenerative capacity of mesenchymal stem cell therapies. To counteract this challenge, cell-free treatments such as specialized exosome-based therapies present a promising regenerative option capable of circumventing obstacles posed by toxic metals.

## 4. Discussion

### 4.1. Environmental Toxic Contamination as a Driver of DFU Development: An Epidemiological and Homeostatic Paradigm

**Figure 2:**
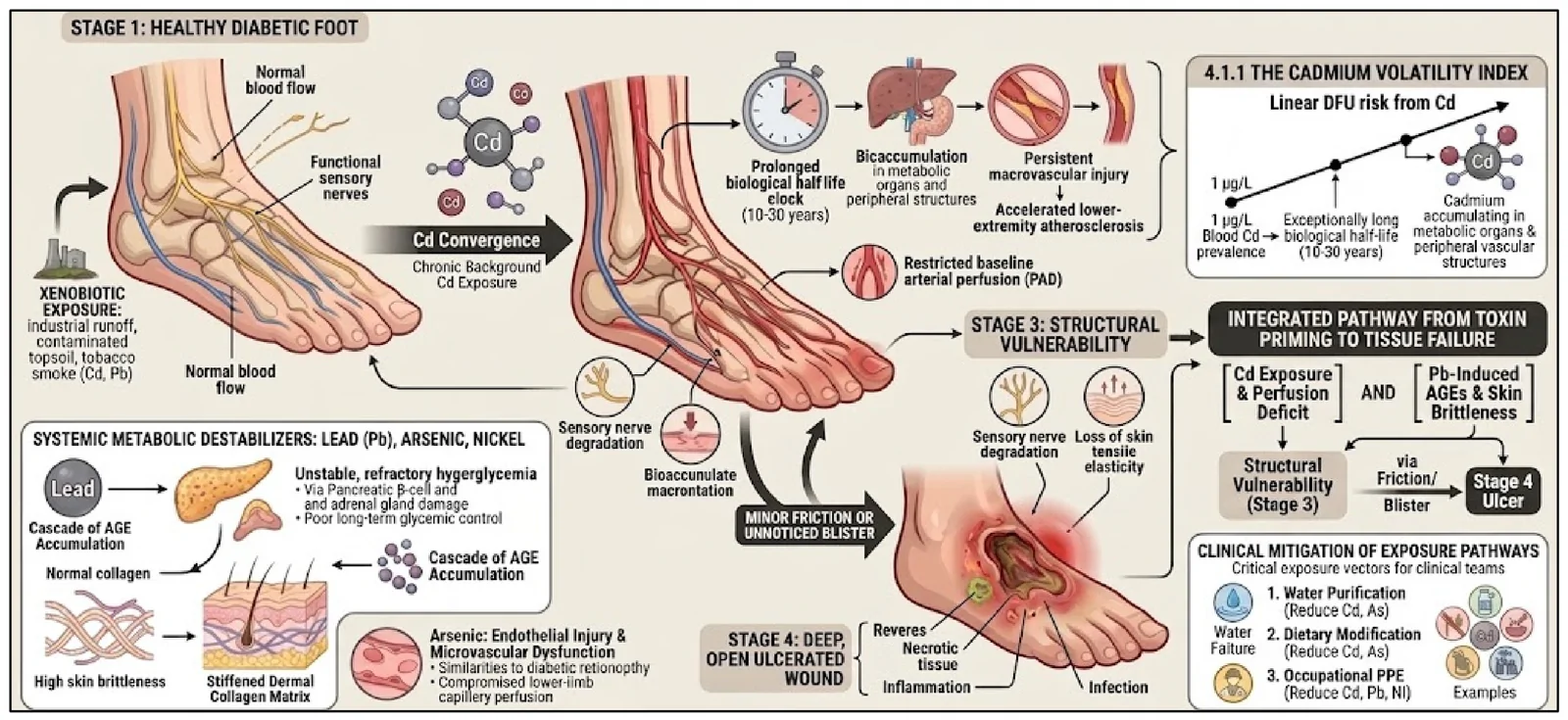
An epidemiological and homeostatic paradigm of DFU development: Environmental toxic contamination as a driver.

Scientific evidence shows that to fully understand how chronic, low-level exposure to environmental toxicants initiates diabetic foot ulcer development, clinical medicine must transition from localised biomechanical models to an integrated environmental-metabolic paradigm, as illustrated in Figure 2. Traditional risk profiles emphasize mechanical shear stress, structural foot deformities, and prolonged tissue pressure. However, epidemiological synthesis reveals that non-essential toxic heavy metals act as foundational primers, undermining systemic tissue resilience long before physical trauma occurs.

#### 4.1.1. The Cadmium Volatility Index

Clinical epidemiological evidence indicates that among the xenobiotics analyzed, cadmium shows a clear linear dose–response relationship with the onset of diabetic foot ulcers. Human cohort data demonstrate that for every 1 µg/L increase in blood cadmium concentration, there is a 64% increase in the prevalence of active foot ulcers, establishing this metal as an independent and clinically observable predictor of tissue failure. The main physiological reason for this strong correlation is the exceptionally long biological half-life of cadmium in the human body, lasting 10 to 30 years, along with its specific accumulation in metabolic organs and peripheral vascular structures [42]. In patients with diabetes mellitus, chronic background exposure to cadmium via industrial runoff, contaminated topsoil, or tobacco smoke induces persistent, subclinical macrovascular injury [43,44]. This exposure significantly accelerates lower-extremity atherosclerotic changes and peripheral arterial disease, systematically restricting baseline arterial perfusion to the distal foot long before an ulcer manifests clinically [45].

#### 4.1.2. Lead and Metalloid-Induced Homeostatic Destabilization

Concurrently, based on cross-sectional human observational data, the elevated blood lead levels documented in diabetic foot ulcer cohorts confirm that chronic lead exposure disrupts the metabolic and microvascular homeostatic baseline [46]. Within a Systems Thinking framework, lead acts as a systemic metabolic destabilizer rather than an isolated toxin [47]. Chronic lead exposure directly impairs pancreatic *β*-cell function and damages adrenal glandular tissue, which clinically manifests as unstable, refractory hyperglycemia and poor long-term glycemic control [47]. This systemic glucose instability triggers a cascade of advanced glycation end-product accumulation, stiffening the dermal collagen matrices of the foot and making the overlying skin highly brittle [48]. Furthermore, when exposed to ambient environmental mixtures containing metalloids such as arsenic or nickel, this pre-existing metabolic strain is compounded by microvascular autoregulatory failure [38,49]. Arsenic, for instance, drives systemic endothelial injury and microvascular dysfunction, mirroring its established role in advanced diabetic retinopathy, which significantly compromises lower-limb capillary perfusion [50,51]. Consequently, the diabetic foot is rendered structurally vulnerable: sensory nerves undergo subclinical degradation, and the skin loses its tensile elasticity [52]. Under these deteriorated conditions, minor mechanical friction or an unnoticed blister rapidly progresses into a deep, open ulcerated wound.

#### 4.1.3. Clinical Thresholds and Exposure Pathways

Table 4 presents a systematic summary of the heavy metal concentration ranges commonly documented in the epidemiological and clinical studies included in this analysis, juxtaposed with established clinical reference values. Moreover, in order to augment the translational and practical applicability of these results to preventive clinical practice, the principal pathways of human exposure are elucidated. It is imperative to comprehend these specific environmental, occupational, dietary, and lifestyle exposure routes. By pinpointing these origins, interdisciplinary diabetic foot management teams can integrate focused environmental screening and exposure mitigation measures such as local water purification, dietary modifications, or occupational personal protective equipment into standard prevention protocols for diabetic populations at high risk.

#### 4.1.4. The Biological Paradox of Essential Transition Metals: Iron, Cobalt, and Manganese

This review mainly discusses the harmful effects of non-essential environmental toxicants (including cadmium, lead, and arsenic). However, a complete understanding of DFU development requires comparing these harmful substances with other important heavy metals in biology. Unlike cadmium and lead, which do not have any known physiological role and only disrupt metabolism, essential transition metals like iron, cobalt, and manganese have a dual biological nature. They are crucial for maintaining normal cellular balance, but when imbalanced in the diabetic environment, they can significantly affect the healing process of wounds.

- Iron (Fe) and Ferroptosis-Driven Oxidative Stress: Iron is essential for oxygen transport, respiration, and angiogenesis. Yet, in diabetic microvascular dysfunction, iron plays a destructive role at the local tissue level. Reduced blood flow in lower limbs and leaky microcapillaries lead to the release of excess iron from erythrocytes into the wound bed. This excess iron triggers the production of harmful hydroxyl radicals through the Fenton reaction, leading to oxidative stress. Additionally, the iron buildup causes ferroptosis, a form of cell death, in dermal fibroblasts and endothelial cells, which worsens inflammation and delays DFU healing.
- Cobalt (Co) and Neurological Maintenance: Cobalt, unlike lead, is essential for the structure of cobalamin (Vitamin B12) and maintaining nerve myelination. Adequate cobalt levels are necessary to protect against diabetic neuropathy progression. Cobalt also helps stabilize hypoxia-inducible factor 1-alpha (HIF-1α) to support new blood vessel growth in the diabetic foot.
- Manganese (Mn) and Mitochondrial Defense: Manganese is vital for enzymes that aid in cellular defense and extracellular matrix repair, especially Manganese Superoxide Dismutase (MnSOD). MnSOD protects cells from superoxide radicals, which are increased in diabetes and toxic metal exposure. Maintaining proper manganese levels safeguards mitochondrial function in keratinocytes, crucial for wound healing.

In conclusion, while removing non-essential toxicants like cadmium and lead is important, managing essential heavy metals such as iron, cobalt, and manganese is complex. Since these metals are essential for wound healing, treatments should focus on restoring local balance, like reducing excess iron while supporting cobalt and manganese pathways—instead of only using broad chelation therapies.

### 4.2. Molecular Pathogenesis and Cross-Phase Tissue Progression: The Cascade of Cellular Failure

**Figure 3:**
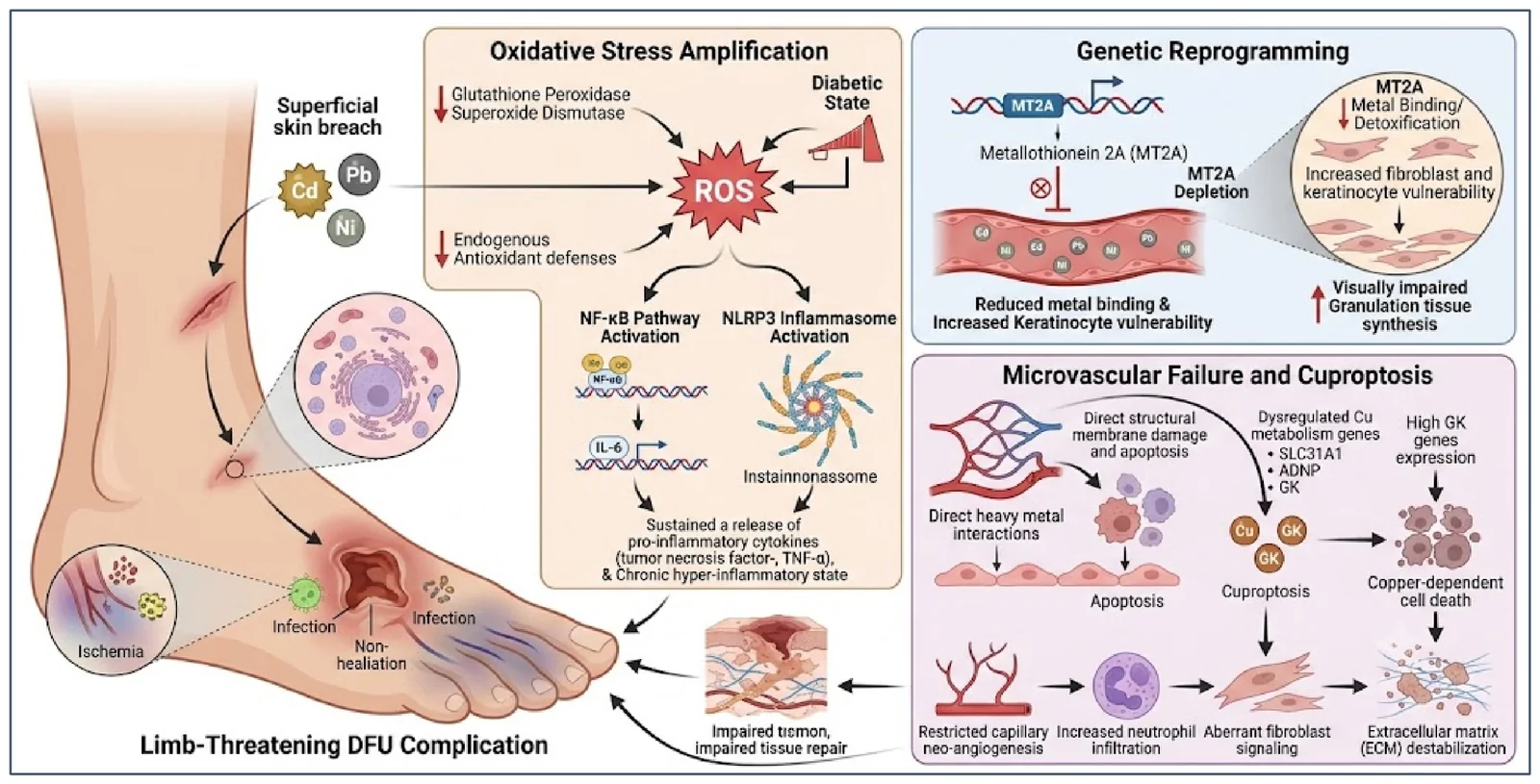
Diabetic Foot Ulcer Progression: The impact of Heavy Metal Toxicity.

While epidemiological data establishes the overall clinical risk, studies conducted in vivo and in vitro suggest that the progression of a diabetic foot ulcer from a superficial skin breach to a deep, non-healing complication is driven by a complex network of heavy metal-induced cellular and genetic disruptions (see Figure 3). Findings from toxicological studies using laboratory models show that non-essential metals (cadmium, lead, and nickel) intersect at a common pathway of cellular damage marked by severe oxidative stress and sustained, chronic inflammatory signalling [53].

#### 4.2.1. Oxidative Stress Amplification and Inflammasome Locking

At the cellular level, experimental cell-line studies demonstrate that heavy metals trigger extensive intracellular damage by dramatically increasing the production of reactive oxygen species while simultaneously disrupting endogenous antioxidant defense systems, such as glutathione peroxidase and superoxide dismutase [54]. In simulated diabetic microenvironments, this heavy-metal-driven oxidative wave acts as a powerful inflammatory amplifier [55]. The prolonged accumulation of free radicals triggers the continuous activation of two primary inflammatory pathways: the Nuclear Factor kappa B (NF-ĸB) and the NLRP3 inflammasome cascades [56].

This molecular activation initiates a sustained, dysregulated release of pro-inflammatory cytokines, specifically interleukin-6 (IL-6) and tumour necrosis factor-alpha (TNF-α), into the surrounding tissue matrix [57]. The persistent presence of these cytokines perpetuates the diabetic foot ulcer wound environment in a chronic, unresolved hyper-inflammatory state [58]. Consequently, the ulcer’s natural progression from the inflammatory phase to the essential proliferative phase of dermal healing is impeded, resulting in a persistently open wound bed highly susceptible to polymicrobial biofilm formation [59].

#### 4.2.2. Genetic and Microvascular Reprogramming

In addition to exacerbating generalized inflammation, heavy metal toxicity induces substantial genetic transcriptional repression and vascular apoptosis within the wound tissue matrix:

- Metallothionein 2A (MT2A) Depletion: In vivo wound healing models indicate that a critical mechanism contributing to localized tissue failure is the marked reduction of MT2A expression within cutaneous wound sections. As MT2A serves as the primary endogenous protein responsible for metal binding, heavy-metal detoxification, and free-radical scavenging, its genetic suppression renders dermal fibroblasts and keratinocytes highly vulnerable to heavy-metal-induced oxidative injury, thereby impairing their capacity for new granulation tissue synthesis [58].
- Angiogenic Failure and Cuproptosis: At the microvascular level, mechanistic studies reveal that heavy metals induce direct structural membrane damage and trigger apoptosis in endothelial cells, substantially restricting the capacity for capillary neo-angiogenesis [60]. This microvascular compromise is further exacerbated by the dysregulation of copper (Cu) metabolism genes, including *SLC31A1*, *ADNP*, and *GK*. Pre-clinical models suggest that high tissue expression of the *GK gene* triggers cuproptosis (copper-dependent, programmed cell death), which reconfigures local fibroblast communication networks [61]. This molecular disruption reorients the wound microenvironment toward heightened neutrophil infiltration and aberrant fibroblast signaling, ultimately destabilizing the structural extracellular matrix and exacerbating lower-limb tissue ischemia [62].

### 4.3. Restorative Therapeutics, Advanced Dressing Technologies, and Detoxification: Overcoming Environmental Blocks

**Figure 4:**
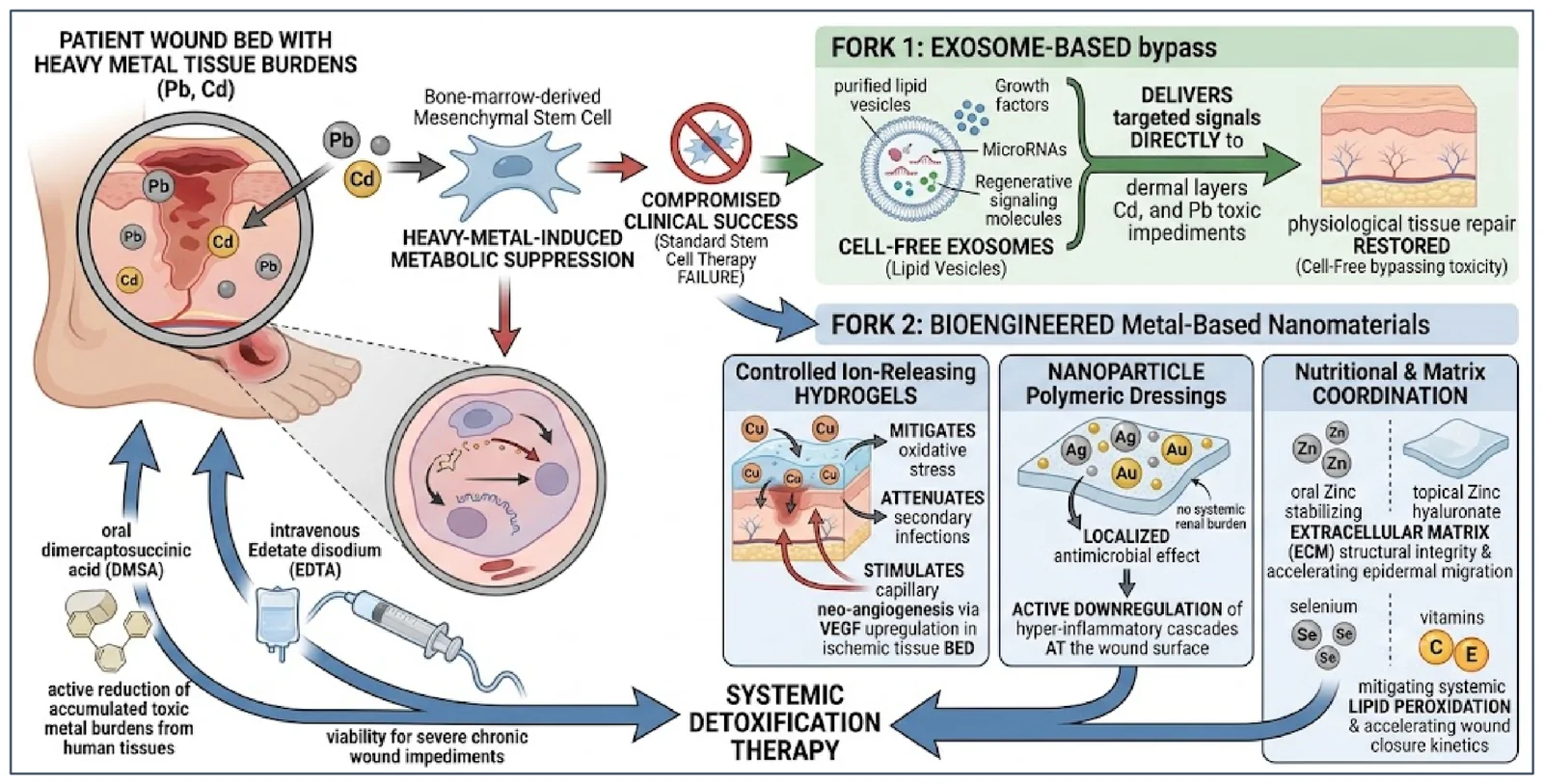
Integrated pathway: heavy metals, advanced wound care and regenerative medicine.

The significant interaction between heavy metal tissue burdens and advanced wound care interventions has profound implications for clinical practice and the advancement of personalized regenerative medicine (shown in Figure 4). Analysis of the clinical outcomes from the 32 included studies reveals that systemic therapeutic approaches, such as targeted oral zinc supplementation and systemic EDTA chelation, are effective interventions that reduce systemic toxic burdens, stabilize extracellular matrix structural integrity, and restore baseline metabolic parameters.

Expanding on these well-established clinical findings, several emerging potential therapeutic strategies resulting from our mechanistic synthesis propose targeted localized interventions to mitigate environmental toxicity within the wound site. A crucial observation in modern regenerative medicine indicates that heightened tissue levels of lead and cadmium directly hinder stem cell metabolic function. This heavy-metal-induced metabolic suppression significantly undermines the efficacy of standard bone-marrow-derived mesenchymal stem cell therapies, as the transplanted cells are quickly compromised by toxic metals accumulated in the patient’s wound site.

#### 4.3.1. The Cell-Free Regenerative Bypass

To address this clinical limitation, advanced cell-free therapies specifically tailored exosome-based treatments have emerged as a compelling alternative. As cell-free exosomes consist of purified lipid vesicles carrying growth factors, microRNAs, and regenerative signaling molecules, they lack the active cellular machinery susceptible to deactivation or toxicity from accumulated heavy metals [63]. This inherent structural robustness enables exosomes to effectively deliver targeted regenerative and tissue-remodelling signals directly to the damaged dermal layers, by passing localized toxic metal impediments to restore physiological tissue repair [64].

#### 4.3.2. Bioengineered Metal-Based Nanomaterials

Furthermore, clinical data emphasize the significant role of advanced bioengineered metal-ion delivery systems and bioactive polymers developed to mitigate local environmental toxicity and enhance structural restoration. Recent in-depth analyses of fundamental wound healing processes illuminate that addressing the persistent inflammatory phase, often extended by oxidative stress induced by heavy metals, necessitates dynamic, multifaceted interventions:

- Advanced Therapeutic Hydrogels: Injectable, multi-crosslinked therapeutic hydrogels engineered for the controlled and sustained release of copper ions present a dual therapeutic advantage. These biomaterials effectively attenuate local microvascular oxidative stress, thereby mitigating the risk of secondary infections, and concurrently stimulate rapid capillary neo-angiogenesis through the upregulation of vascular endothelial growth factor in ischemic tissues. Recent advancements in soft-matter bioengineering have facilitated the development of sophisticated, multifunctional dressings specifically adapted for the challenging diabetic wound microenvironment. For example, the innovation of specialized antibiofilm and antioxidative hydrogel dressings offers an active defense mechanism capable of simultaneously neutralizing heavy-metal-induced reactive oxygen species and disrupting the dense polymicrobial biofilms frequently observed in chronic diabetic ulcers [65]. Furthermore, contemporary material design prioritizes the incorporation of degradable hydrogels. These environmentally responsive matrices function as transient extracellular scaffolds, supporting sustained cellular migration and proliferation, while safely degrading synchronously with neo-tissue formation to avert secondary physical trauma during dressing removal [65,66].
- Nanoparticle Polymeric Dressings: Advanced polymeric wound dressings integrating non-toxic therapeutic silver and gold nanoparticles represent a significant advancement in the localized management of diabetic foot ulcers. These dressings demonstrate potent localized antimicrobial efficacy and effectively mitigate hyper-inflammatory responses directly at the wound site, circumventing the introduction of systemic toxins or imposing an additional renal detoxification burden on the patient. The expanded application of bioactive agent-loaded polymers signifies a highly versatile frontier in diabetic wound management. As comprehensively evaluated in the recent literature [67], these engineered polymeric systems can be precisely customized to function as multi-drug delivery vehicles. They concurrently deliver localized heavy metal chelators, potent reactive oxygen species (ROS) scavengers, and pro-angiogenic factors directly into the compromised wound bed, systematically addressing the environmental impediments to tissue regeneration.
- Nutritional and Matrix Coordination: On a systemic level, correcting trace element deficiencies constitutes a fundamental requirement. Targeted oral zinc supplementation and the application of topical zinc hyaluronate dressings effectively stabilize extracellular matrix structural integrity and accelerate epidermal migration [66]. Concurrently, selenium replenishment, when co-administered alongside antioxidant micronutrients such as vitamins C and E, effectively mitigates systemic lipid peroxidation and accelerates overall wound closure kinetics [68].
- Clinical Efficacy of Systemic Detoxification: Finally, for patients presenting with severe, chronic wound-healing impediments driven by extensive heavy metal bioaccumulation, active systemic detoxification is recognized as a viable therapeutic strategy [22]. Clinical data investigating heavy metal chelation therapies utilizing oral dimercaptosuccinic acid (DMSA) or intravenous Edetate disodium demonstrate a clear capacity to reduce accumulated toxic metal burdens from human tissues [69].

In patients presenting with advanced diabetes complications, macrovascular disease, and critical limb ischemia, targeted EDTA chelation therapy has demonstrated significant efficacy in promoting clinical limb salvage and mitigating adverse cardiovascular events [70]. On a molecular level, the removal of these accumulated toxic elements successfully reverses heavy-metal-induced bone marrow suppression, leading to a significant expansion in the population of healthy, active circulating stem and progenitor cells. This detoxification process thus re-establishes the intrinsic healing capacity, transforming previously non-healing, refractory ulcers into responsive, treatable wounds [71].

### 4.4. Strengths and Limitations

The strengths of this systematic review include its robust methodology, characterized by the concurrent application of the validated Newcastle-Ottawa Scale and SYRCLE’s Risk of Bias tool for the comprehensive appraisal of heterogeneous epidemiological and toxicological datasets. Furthermore, the integration of a novel Systems Thinking framework facilitated the effective conceptual integration of environmental toxicology data with molecular diabetic pathways. The study’s limitations include the reliance on observational studies and the inability to establish longitudinal causality across all cohorts. In addition, substantial heterogeneity was observed in exposure assessments across various biological matrices. Moreover, there is a paucity of direct clinical trials evaluating chelation therapy specifically within diabetic foot ulcer populations.

## 5. Conclusions

This systematic review comprehensively analyzes environmental heavy metal exposure as a significant etiological factor contributing to diabetic foot complications, thereby advocating for a reorientation of the clinical paradigm towards an integrated environmental-metabolic model. The synthesized evidence substantiates that chronic bioaccumulation of toxic metals, such as cadmium and lead, markedly elevates ulcer prevalence and accelerates tissue deterioration. This detrimental progression is primarily mediated by pronounced heavy metal-induced oxidative stress, the activation of the NLRP3 inflammasome, and the downregulation of protective genetic pathways, such as MT2A. Conversely, therapeutic strategies, including the rectification of essential trace metal deficiencies and the deployment of advanced metal-based bioengineered dressings or cell-free exosome therapies, offer compelling prospects for re-establishing homeostasis within the wound microenvironment. Ultimately, expanded longitudinal research and the implementation of robust public health screening protocols for environmental toxicant burdens are paramount for enhancing limb salvage rates and optimizing personalized care in vulnerable diabetic populations.

## Figures and Tables

**Table 1:** Methodological quality and risk of bias scores for epidemiological studies (Newcastle-Ottawa Scale).

Author/s (Year)	Study Design	Selection (Max 4★)	Comparability (Max 2★)	Outcome/Exposure (Max 3★)	Total Score	Quality Tier
Xing et al. (2025) [9]	Cross-Sectional	★★★★	★★	★★	8/9	High Quality
Feezan et al. (2025) [34]	Cohort	★★★	★	★★	6/9	Moderate Quality
Hyun et al. (2022) [35]	Retrospective Cohort	★★★★	★	★★	7/9	High Quality
Karim Suri et al. (2026) [36]	Cross-Sectional	★★★	★	★★	6/9	Moderate Quality
Yadav et al. (2019) [37]	Cohort	★★★★	★★	★★	8/9	High Quality
Lee et al. (2024) [38]	Cohort/Tissue Assay	★★★	★	★★	6/9	Moderate Quality
Nakamura et al. (2025) [39]	Cohort	★★★	★	★★	6/9	Moderate Quality
Momen-Heravi et al. (2017) [40]	RCT * (Modified NOS)	★★★★	★★	★★★	9/9	High Quality
Yang et al. (2026) [41]	Cohort	★★★	★	★	5/9	Moderate Quality

★ (Stars): Represent the quality score awarded based on the Newcastle-Ottawa Scale (NOS) criteria, * (Asterisk): Indicates that the study design was a Randomized Controlled Trial (RCT).

**Table 2:** The SYRCLE tool for in vivo models.

Evaluation Domain	Risk Level	Percentage (%) of Studies	Primary Methodological Observation
Sequence Generation (Selection Bias)	Low Risk	78%	Most studies clearly stated the randomization methods used to allocate animals to metal exposure/control groups.
Allocation Concealment (Selection Bias)	Unclear Risk	85%	Sealed or centralized allocation pipelines were rarely detailed in the laboratory protocols.
Housing and Randomization (Performance Bias)	Low Risk	90%	Environmental control matrices (temperature, lighting, dietary baselines) were well-standardized across groups.
Blinding of Investigators (Performance Bias)	High Risk	65%	Investigators delivering heavy metal treatments (e.g., via drinking water or gavage) were rarely blinded.
Random Outcome Assessment (Detection Bias)	Unclear Risk	70%	Specific details on whether wound measurement devices or tissue sections were randomized during assessment were omitted.
Blinding of Outcome Assessors (Detection Bias)	Low Risk	60%	Histopathological and genetic analysts were frequently blinded to the specific heavy metal concentration exposure arm.
Incomplete Outcome Data (Attrition Bias)	Low Risk	92%	Animal exclusions, operational attritions, and post-exposure fatalities were explicitly reported with precise group numbers.
Selective Outcome Reporting (Reporting Bias)	Low Risk	88%	Extracted data matched the expected biochemical, molecular, and histological markers promised in the experimental abstracts.

**Table 3:** Matrix of trace element impacts, molecular mechanisms, and DFU therapeutic interventions.

Element	Clinical Association and Impact on DFU	Primary Mechanistic Role	Therapeutic Interventions
Cadmium	Increased Prevalence, Increased tissue severity, and chronic non-healing ulcers	Drives ROS production, downregulates tissue MT2A expression	Systemic chelation therapy; exposure reduction
Lead	Increased Prevalence, poor glycemic control, increased macrovascular risk	Drives neuroinflammation via NF-ĸB/NLRP3 activation	Systemic chelation (e.g., EDTA); exposure mitigation
Zinc	Deficiency correlates with Increased DFU incidence and severity	Regulates extracellular matrix structural maintenance	Oral zinc supplementation; topical zinc hyaluronate
Copper	Dual role: Systemic deficiency impairs healing; local excess is toxic	Regulates angiogenesis and cuproptosis-driven fibroblast crosstalk	Ion-releasing therapeutic hydrogels
Silver	Non-toxic at therapeutic levels; accelerates wound closure	Potent local antimicrobial activity; dismantles local bacterial burden	Silver-impregnated polymeric dressings.
Gold	Biologically inert; non-toxic at therapeutic levels; accelerates wound closure	Modulates local hyper-inflammation and reduces oxidative stress (no direct antimicrobial activity)	Gold nanoparticle-embedded biomaterial matrices.

**Table 4:** Reference values, reported concentrations, and major exposure pathways for heavy metals implicated in diabetic foot complications.

Heavy Metal/Toxin	Biological Matrix	Reported Concentration Range in Diabetic/DFU Cohorts	Standard Clinical Reference Value	Primary Sources and Pathways of Exposure
Cadmium (Cd)	Whole Blood	1.5–5.2 µg/L	<1.0 µg/L (non-smokers)	Lifestyle: Active and passive tobacco smoke. Environmental: Industrial runoff, contaminated topsoil. Dietary: Root vegetables and grains grown in contaminated soil. Occupational: Smelting, battery manufacturing.
	Urine	1.0–3.8 µg/g creatinine	<1.0 µg/g creatinine	
Lead (Pb)	Whole Blood	3.5–18.0 µg/dL	<5.0 µg/dL	Occupational: Construction, battery recycling, mining, radiator repair. Environmental: Legacy leaded-paint/dust, contaminated drinking water (ageing plumbing infrastructure). Dietary: Contaminated herbal supplements or spices.
Arsenic (As)	Urine (Speciated)	40.0–95.0 µg/L	<35.0 µg/L	Environmental: Contaminated groundwater and potable drinking water (endemic in specific geographic regions). Dietary: Rice, specific seafood/shellfish. Occupational: Agricultural pesticides and herbicides, wood preservation.
Nickel (Ni)	Whole Blood	3.0–9.2 µg/L	<2.0 µg/L	Occupational: Stainless steel welding, electroplating, and metal refining. Environmental: Ambient air pollution near industrial zones. Dietary: High-nickel foods (cacao, certain nuts, legumes) and leaching from certain cookware.
	Urine	4.5–12.0 µg/L	<5.0 µg/L	

## Data Availability

No new data were created or analyzed in this study. Data sharing is not applicable to this article.

## Abbreviations

The following abbreviations are used in this manuscript:

AAS	Atomic Absorption Spectroscopy
As	Arsenic
Cd	Cadmium
Cu	Copper
DFU	Diabetic foot ulcer
DMSA	Dimercaptosuccinic acid
EDTA	Edetate disodium
Hg	Mercury
HR	Hazard Ratios
ICP-MS	Inductively Coupled Plasma Mass Spectrometry
IL-6	Interleukin-6
MT2A	Metallothionein 2A
NO	Nitric oxide
NOS	Newcastle-Ottawa Scale
OR	Odds Ratio
PAD	Peripheral arterial disease
Pb	Lead
PECOS	Population, Exposure, Comparator, Outcome, and Study Design
RR	Relative Risks
